# A Multidimensional Approach to the Study of Emotion Recognition in Autism Spectrum Disorders

**DOI:** 10.3389/fpsyg.2015.01954

**Published:** 2015-12-24

**Authors:** Jean Xavier, Violaine Vignaud, Rosa Ruggiero, Nicolas Bodeau, David Cohen, Laurence Chaby

**Affiliations:** ^1^Département de Psychiatrie de l’Enfant et l’Adolescent, APHP, Groupe Hospitalier Pitié-SalpêtrièreParis, France; ^2^Institut des Systèmes Intelligents et Robotique, CNRS UMR 7222, Paris DescartesParis, France; ^3^Dipartimento di Psicologia, Seconda Università Degli Studi Di NapoliCaserta, Italie; ^4^Institut de Psychologie, Université Paris Descartes, Sorbonne Paris CitéBoulogne-Billancourt, France

**Keywords:** autism spectrum disorders, facial emotion, vocal emotion, multimodal integration, eye tracking, language comorbidity, fine motor skills, neuro-visual skills

## Abstract

Although deficits in emotion recognition have been widely reported in autism spectrum disorder (ASD), experiments have been restricted to either facial or vocal expressions. Here, we explored multimodal emotion processing in children with ASD (*N* = 19) and with typical development (TD, *N* = 19), considering uni (faces and voices) and multimodal (faces/voices simultaneously) stimuli and developmental comorbidities (neuro-visual, language and motor impairments). Compared to TD controls, children with ASD had rather high and heterogeneous emotion recognition scores but showed also several significant differences: lower emotion recognition scores for visual stimuli, for neutral emotion, and a greater number of saccades during visual task. Multivariate analyses showed that: (1) the difficulties they experienced with visual stimuli were partially alleviated with multimodal stimuli. (2) Developmental age was significantly associated with emotion recognition in TD children, whereas it was the case only for the multimodal task in children with ASD. (3) Language impairments tended to be associated with emotion recognition scores of ASD children in the auditory modality. Conversely, in the visual or bimodal (visuo-auditory) tasks, the impact of developmental coordination disorder or neuro-visual impairments was not found. We conclude that impaired emotion processing constitutes a dimension to explore in the field of ASD, as research has the potential to define more homogeneous subgroups and tailored interventions. However, it is clear that developmental age, the nature of the stimuli, and other developmental comorbidities must also be taken into account when studying this dimension.

## Introduction

Autism spectrum disorder (ASD) is a heterogeneous group of neurodevelopmental disorders. Before the Diagnostic and Statistical Manual of Mental Disorders-5 (DSM-5), which was the manual that officially labeled this term as a diagnostic category, ASD was used as a common clinical term that referred to the pervasive developmental disorders (PDD), as described in the DSM IV-TR [American Psychiatric Association (APA), 2000] and the ICD-10 (10th International Classification of Diseases). Successive redefinitions of autism diagnostic criteria have not succeeded in constraining complexity and comorbidity in autism. A multidimensional point of view should encompass the issues posed by this categorical approach. From this perspective, child development is shaped by the interaction between several dimensions (e.g., language, motor, cognition, emotion; Xavier et al., 2015). In the present study, we focus on emotion recognition, taking into account uni- and multimodal stimuli as well as other developmental comorbidities that are not included in ASD criteria but that are frequently associated with ASD, specifically language, fine motor, and neuro-visual skills.

Since Kanner’s (1943) first clinical description of autism, problems related to emotion processing have been seen as a hallmark symptom of the disorder. However, the status of emotion impairments remains, until now, uncertain. Research that examines emotion recognition in ASD has been limited by an over-focus on the visual modality, specifically the recognition of emotion in facial expressions. Several studies have reported that children with ASD display deficits in this ability (Downs and Smith, 2004; Sinzig et al., 2008; Harms et al., 2010; Uljarevic and Hamilton, 2013).

However, several studies have failed to find significant differences in emotion recognition tasks when comparing children with ASD and controls (Braverman et al., 1989; Ozonoff et al., 1990; Prior et al., 1990; Buitelaar et al., 1999; Grossman et al., 2000; Pelphrey et al., 2002; Castelli, 2005; Jones et al., 2011). Additionally, the nature of the deficits in emotion processing has been discussed, but with limited agreement among experts. The difficulties that children with ASD have with emotion processing may relate to their inability to recognize specific emotions, such as fear and disgust (Humphreys et al., 2007; Wallace et al., 2008), anger (Ashwin et al., 2006), or sadness (Corden et al., 2008; Wallace et al., 2008). At the same time, other authors have failed to find any deficits in the recognition of negative emotions (Lacroix et al., 2009). Overall, emotion recognition deficits do not appear to be universal in ASD, as reflected by heterogeneous performance across children and across tasks (for a review, see Nuske et al., 2013). Studies using eye-tracking technology have examined the scanning of emotional faces in children with ASD and have found that they globally spend less time on core features (i.e., eyes, nose, and mouth) as compared to typically-developing (TD) children (e.g., de Wit et al., 2008). However, more recent studies have found that deficits in emotion recognition cannot be fully explained by differences in face scanning, as children with ASD showed no particular differences in time fixation on faces (Sawyer et al., 2012) or showed normative pupillary reactions to emotion expressed by familiar people (Nuske et al., 2014). Overall, the idea of gaze abnormalities in ASD, including less time spent focusing on emotional cues (e.g., facial expressions), remains controversial (for a review, see Guillon et al., 2014; Yi et al., 2014).

Understanding emotional states in real life involves processing a variety of cues that include verbal content, non-verbal cues (e.g., postures), non-verbal vocalization, and affective prosody (Chaby et al., 2012). The recognition of emotion from vocalizations, which appears to be the auditory equivalent of facial emotion recognition, has been less studied, with limited and inconsistent findings. A few studies have found that, compared to typically developing children, children with ASD demonstrate impaired auditory emotion expression recognition skills (Philip et al., 2010; Charbonneau et al., 2013). On the other hand, others have found no evidence of a fundamental deficit in ASD (Baker et al., 2010; Jones et al., 2011).

According to the “weak central coherence theory,” which was conceptualized by Happe and Frith (2006) and corresponds to the difficulties that children with ASD have when synthesizing stimuli into a coherent whole, children with ASD have impairments in multisensory processing (Bebko et al., 2006; Magnée et al., 2008; Mongillo et al., 2008; Megnin et al., 2012; Russo et al., 2012; Collignon et al., 2013). Charbonneau et al. (2013) noted that both ASD and TD children benefited from exposure to bimodal information, but to a lesser extent in the ASD group than the TD group. Vannetzel et al. (2010) explored the processing of neutral and emotional human stimuli (in the auditory, visual, and multimodal channels) in children with PDD-Not Otherwise Specified (NOS), when compared to TD children. The PDD-NOS group experienced difficulties with processing emotional stimuli, particularly in the visual modality. They also more easily identified happy, rather than angry or neutral, faces, and vocalizations. The children with PDD-NOS used the multimodal channel to compensate for their unimodal deficits. Similarly, Jones et al. (2011) studied emotion recognition abilities by using a combination of visual and auditory tasks for two groups of adolescents, with and without ASD. They found that IQ had a large and significant effect on performance, but they also found no evidence of a fundamental deficit with emotion recognition in the adolescents with ASD. The discrepancies in the conclusions of the different studies that have been described above are underpinned by several factors: (1) the use of different experimental designs (e.g., only visual tasks) and data analyses (e.g., only univariate analyses); (2) the sizes and characteristics of the samples; (3) the heterogeneity among ASD patients in terms of developmental course requiring the consideration of developmental age when comparing to TD children; and (4) the heterogeneity among ASD patients in terms of co-occurring impairments that *per se* could affect emotion recognition such as visual-motor impairments or developmental coordination disorder (DCD) for visual tasks and language deficits for auditory tasks.

The aim of the current study was to explore unimodal and multimodal emotion processing in children with ASD by comparing them to TD children and taking into account other developmental comorbid factors such as language, fine motor, and neuro-visual impairments (using eye-tracking technology for this last dimension). In order to understand the nature of the heterogeneity and discrepancies in the results between studies mentioned above, we chose to take into account comorbid factors and to assess a potential age effect by including a sample of children with ASD in a wide age range (6–13). We hypothesized that: (1) consistent with the literature, we expect to find significant heterogeneity in children with ASD, with regard to their emotional identification skills; (2) in comparison to the TD group, children with ASD will have difficulties processing emotional information, which will be most prevalent in the unimodal channel (visual or auditory) and partially alleviated in the multimodal condition; (3) because ASD presents atypical eye movements and fine motor skills, we expect impairments in the visual modality to be larger in children with ASD; and (4) similarly, we expect that the subgroup of patients with language disorders will be more impaired in regards to unimodal auditory stimuli.

## Materials and Methods

### Participants

A total of 19 children with ASD and 19 typically developing children (14 boys and 5 girls in each group) participated in this study. ASD was used as a common clinical term that referred to the PDD that were described in the ICD-10 classification system (World Health Organization, 1993). Two trained child psychiatrists (JX and DC) clinically assessed the children with ASD (mental age range = 5.8–13.3 years, *M* age = 7.74 years, *SD* = 2.51). In reference to the PDD diagnostic category, 5 children satisfied the diagnostic criteria for autism, and 14 children satisfied the criteria for atypical autism (i.e., presence of abnormal or impaired development before the age of three and abnormalities in reciprocal social interactions or in communication without fulfilling the full criteria for autism).

Children with ASD were recruited via the Child and Adolescent Psychiatry department at Pitié-Salpétrière Hospital and the University Pierre-et-Marie-Curie in Paris, France. **Table 1** summarizes the socio-demographic and clinical characteristics of the patients. Each child with ASD was individually matched, according to developmental age, with a healthy typically developing child using chronological age (TD: age range = 6–13 years, Mean age = 8.84 years, *SD* = 1.79). The 19 TD children were recruited via a local primary school. The study was conducted in accordance with the hospital’s Research Ethics Board regulations. After being fully informed about the study, parents or legal caregivers provided written consent.

**Table 1:** Main characteristics of children with autism spectrum disorder (ASD; *N* = 19).

Chronological age, mean (±SD)	9.95 (1.75)
Male/Female	14/5
Socio-Economic Status: high/middle/low	10/5/4

*Children’s Global Assessment Scale, mean (±SD)*	
IQ score	78.5 (21.02)
Mental age (IQ^∗^ age/100)	7.74 (2.51)
ADI-R scores	
Social impairment	10.11 (5.70)
Verbal Communication	7.06 (4.22)
Repetitive interest	2.72 (2.56)
VABS for Children (socialization domain)	
Interpersonal relationships	34.59 (8.39)
Play and leisure time	21.25 (6.61)
Coping skills	15.06 (5.98)
*Total score*	70.63 (18.17)

*Diagnosis (ICD-10)*	
Atypical autism	14
Autism disorder	5
Developmental disorder of speech and language	9
Developmental Coordination Disorder (DCD)	12


*VABS, Vineland Adaptive Behavior Scales; WISC, Wechsler Intelligence Scale for Children.*

During a 1-week period of clinical testing, each child was given a series of clinical assessments: the Autism Diagnostic Interview-Revised (ADI-R) was used to score autism core symptoms (Lord et al., 1994); the Children’s Global Assessment Scale (CGAS; DSM-5, 2013) was used to score global severity; and Vineland Adaptive Behavior Scales were used to score psychosocial/adaptive functioning (Sparrow et al., 1984). The cognitive quotient was ascertained by using the WISC-IV (Wechsler Intelligent Scale for Children-IV), the WPPSI (Wechsler Preschool and Primary Scale of Intelligence), or the KABC-II (Kaufman Assessment Battery for Children, second edition). Developmental age was calculated on this basis to match patients with TD children while taking into account possible intellectual deficits. To evaluate the existence of clinical developmental comorbidities, patients also received (i) a speech and language assessment using the ELO Battery (*Evaluation du Langage Oral*: Assessment of Oral Language; Khomsi, 2001) by a speech therapist, and (ii) a psychomotor assessment using the M-ABC (Movement Assessment Battery for Children; Henderson and Sugden, 1992) performed by an occupational therapist.

### Stimuli

**FIGURE 1:**
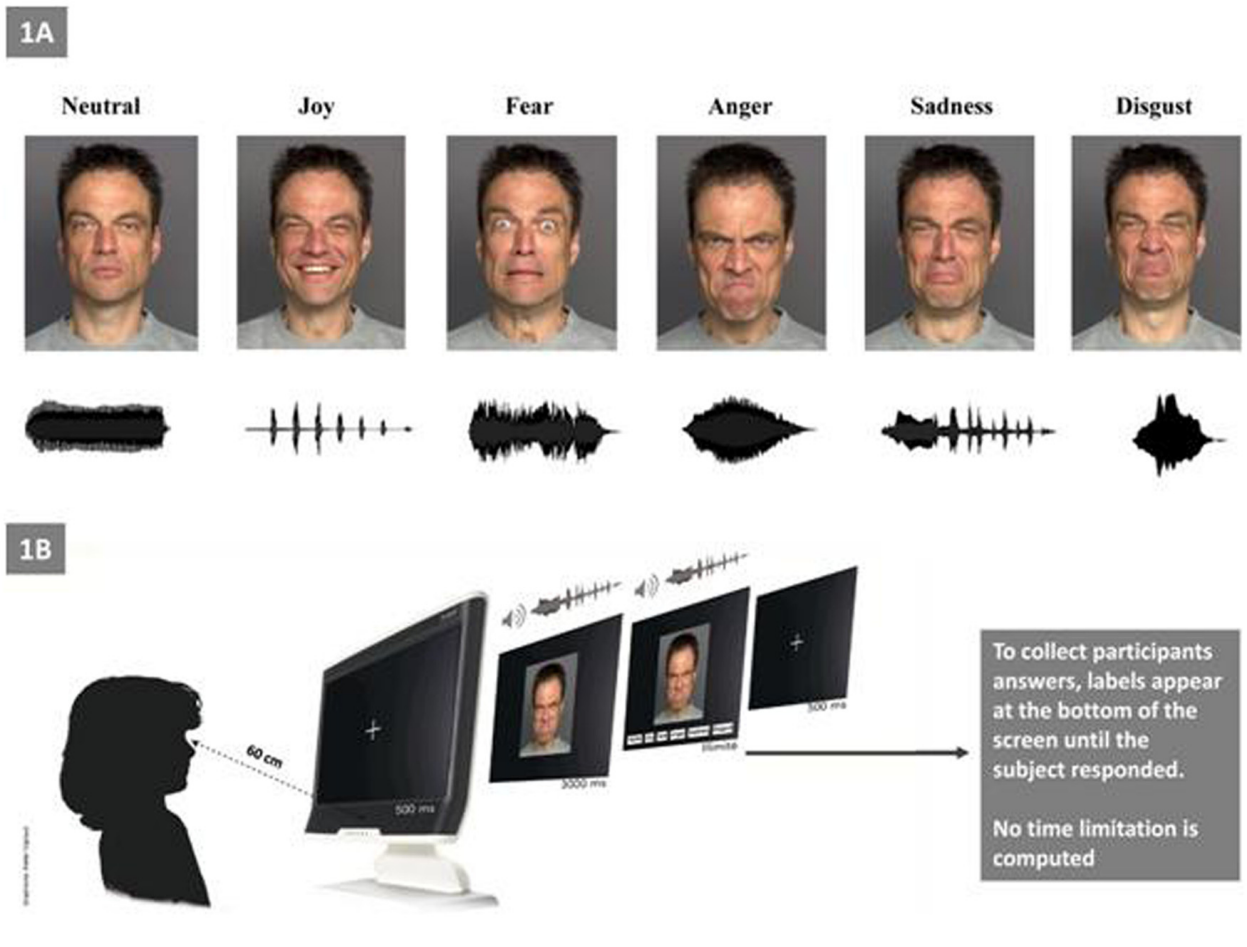
**Experimental setting.** **(A)** Examples of facial and vocal emotional stimuli presentations for each of the six primary emotions (e.g., “Joy” with the outline of a prosodic laugh). **(B)** Experimental procedure.

As described by Luherne-du Boullay et al. (2014), the stimuli used in the present study were categorized as visual, auditory, or bimodal (**Figure 1A**). *Visual stimuli* consisted of pictures of facial expressions that were obtained from the FACES database (Ebner et al., 2010). The faces of six actors (three men, three women) who expressed six facial expressions (joy, fear, anger, sadness, disgust, and neutral) constituted a set of 36 visual stimuli. *Auditory stimuli* included non-verbal affective vocalizations from the Montreal Affective Voices database (Belin et al., 2008), in which actors produced emotional interjections by using the vowel /a/ (cry, laugh, etc.). The voices of six actors (three men, three women) who expressed six vocal expressions (joy, fear, anger, sadness, disgust, and a neutral) constituted a set of 36 auditory stimuli. *Bimodal stimuli* consisted of congruent combinations of an emotional face and an affective vocalization (36 bimodal stimuli).

### Eye-Tracking Apparatus

During tasks with visual and bimodal stimuli, children’s gazes were monitored by using an integrated Tobii T120 eye-tracker (Tobii Technology, Danderyd, Sweden). The T120 eye-tracker is a device that is built into a screen (17-inch) and does not require restriction of the children’s heads, thus allowing them to look at the pictures freely and naturally. The system tracks both of the children’s eyes separately at a rated accuracy of 0.5 degrees and a sampling rate of 120 Hz. A five-point infant calibration was used, and the experiment began after the points were correctly calibrated. By using the Tobii Studio Analysis Software, we evaluated the cumulative number of saccades (as an index of eye movements) and the durations of fixations (as an index of eye stability) on the face. Thus, one area-of-interest (AOI) region was defined for each face image including the whole face. A minimum of 50% valid gaze time was required for the analysis. In the ASD group, seven children were excluded from the eye-tracking analysis, as they did not yield data that were valid for any of the three tasks (e.g., due to excessive movement). A Wilcoxon test comparing the mental age of the 19 vs. 12 subjects remaining was not significant (*W* = 75.5, *p* = 0.122), so we are able to conclude that no bias was introduced by removing the seven patients from the analysis.

### Procedure

Before the experimental procedure, we ensured that all participants were able to understand each basic emotion (i.e., we asked each child to explain with examples of each emotion; for the ASD group this ability was confirmed by their language assessments). Children were tested individually in a single session that lasted approximately 30 min (**Figure 1B**). The experiment consisted of three tasks: visual (facial emotions), auditory (vocal emotions), and bimodal. The bimodal (audio–visual) stimuli consisted in the synchronous and congruent facial and vocal presentation for the same emotion. The experiment was run with E-prime software. After the statement of the set, the eye calibration and the familiarization with the four sample items, the experiment began. Each child was seated approximately 60 cm far from the screen of the eye-tracker. Each trial began with the presentation of a fixation cross (500 ms), which was followed by the presentation of the target stimulus during 3 s (i.e., temporal window for the eye-tracking recording); then, labels appeared at the bottom of the screen until the child responded. During the auditory task, no visual stimuli appears on the screen. Participants were asked to select (by clicking with the computer mouse) one label from a list choice that best described the emotion that was being expressed.

The order of the three tasks was counterbalanced across children, and the order of trials was pseudo-randomized across each task. There was an inter-trial interval of 700 ms, and a resting pause was offered after every ten trials. Correct answers and the eye-tracking data (the number of saccades and fixation durations on the faces in the visual and bimodal tasks) were recorded.

### Statistical Analysis

The data for the present study were Analysed using the statistical program R, version 2.12.2 (R Foundation for Statistical Computing), with two-tailed tests and a 95% confidence level. Due to the repeated measures design and the forced choice paradigm (six possible answers) that was used in our experiment, we used a Generalized Linear Mixed Model (GLMM; lme4 package) to explore the data. A binomial family was specified in the GLMM model to estimate the log-odds ratio for the corresponding factors in the model. With the exception of the eye tracking experiment, we used the entire sample (19 vs. 19).

In the general analysis, factors included group (ASD vs. TD), emotion (joy, neutral, anger, disgust, fear, or sadness), task (visual, auditory, or bimodal), developmental mental age (DA) and sex (model formulation: number of successes ∼ Task + Emotion + Group + DA + Sex + Random Participant factor). Then, in tasks where gaze was recorded (unimodal visual and bimodal tasks), we performed new analyses by including fixation duration and number of saccades as additional factors in the precedent model. It is not possible to compute the observed power in the case of mixed models. However, a good estimation is to compute the power for a standard regression and consider the true power to be a bit higher due to the repeated measures design. In our case, if we considered three regressors (group, stimulus, and emotion), a sample size of 38, a type I error rate of 0.05, and a large effect size of 0.35 (Cohen, 1988), the power of the regression was 84%. A medium effect size of 0.15 gave a power of 45%, so even if the true power was >50%, our experimental protocol did not allow us to detect small differences.

A secondary multidimensional analysis was conducted in the entire sample for each task separately (i.e., visual, auditory, and bimodal), and then in each group. In the ASD subgroup, we added the following dimensional factors: Vineland total score (VABS), language disorder and DCD to assess whether such comorbidities affected participant scores during the task.

## Results

### General Analysis

**FIGURE 2:**
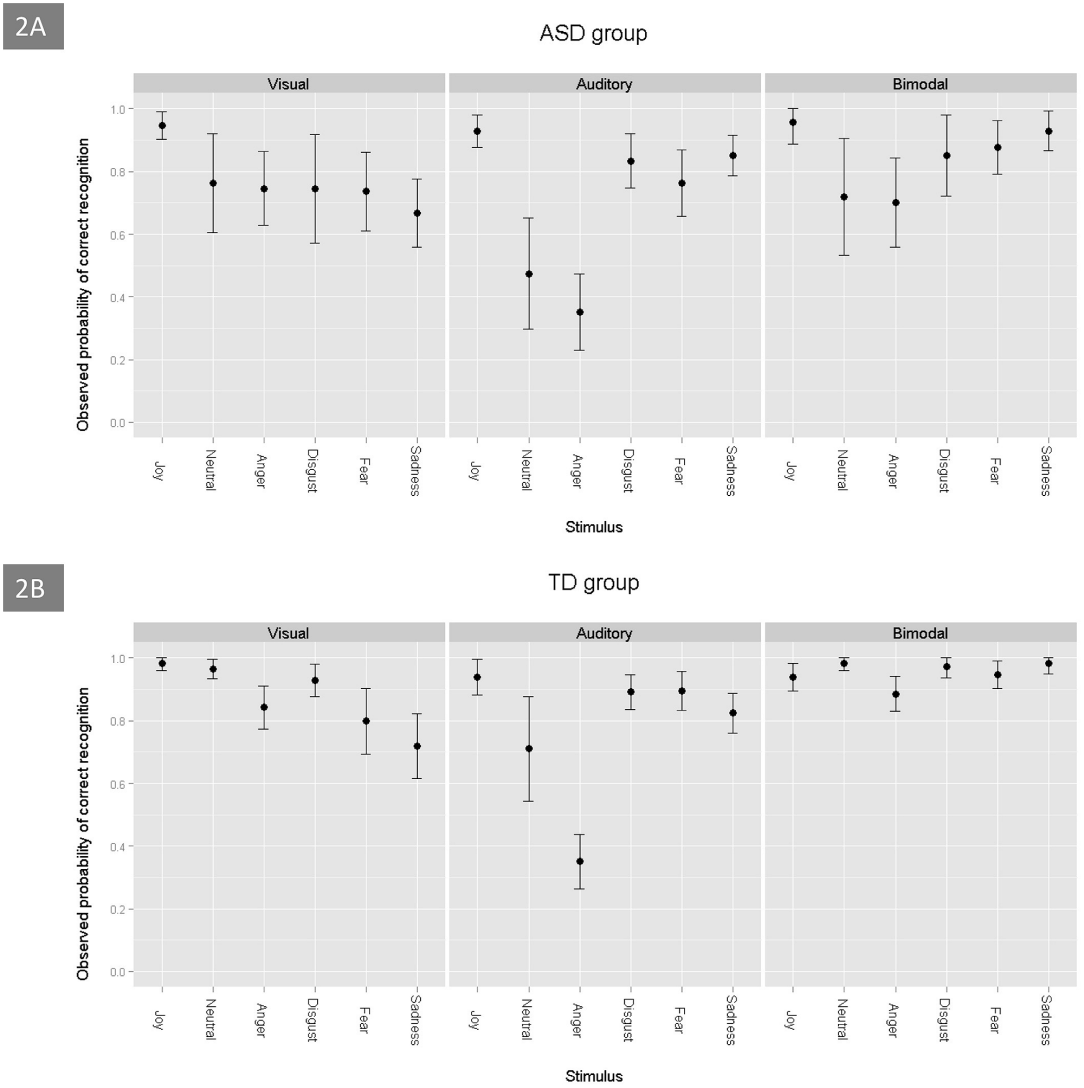
**Probability of correct emotion recognition, as a function of the emotions and tasks, in ASD **(A)** and TD **(B)** children**.

**Figure 2** shows the probabilities of correct answers being obtained by children with ASD and TD control children on the three tasks (visual, auditory, and bimodal) and for each emotion. For the two groups, the probability of success on the bimodal task was greater than on the unimodal visual task (estimate = -0.78, *p* < 0.001), which was greater than that on the unimodal auditory task (estimate = 0.61, *p* < 0.001). The GLMM model revealed that the rate of correct emotion recognition depended on specific emotions: joy was the most easily recognized by the two groups of children, when compared to the emotions of neutral (estimate = -1.96, *p* < 0.001), sadness (estimate = -1.51, *p* < 0.001), anger (estimate = -2.68, *p* < 0.001), fear (estimate = -1.45, *p* < 0.001) and disgust (estimate = -1.13, *p* < 0.001). The ASD group was able to perform the multimodal task. Regardless of the emotion that was involved, their probability to succeed was over 0.7 (**Figure 2A**). Neutral and anger were the most difficult emotions to identify for the ASD group (estimate = -1.76, *p* < 0.001, and estimate = -1.79, *p* < 0.001, respectively). Regarding all of the tasks, we found no significant difference between the results that were obtained by the two groups (estimate = -0.31, *p* = 0.104), but the scores were strongly and positively associated with the children’s developmental age (estimate = 0.27, *p* < 0.001).

When taking into account the combination of the two visual stimuli (visual unimodal and bimodal) and the eye-tracking variables, the ASD group performed worse than the control group (estimate = -0.75, *p* = 0.03). In addition, the discrepancy between the scores of disgust and joy (estimate = -1.27, *p* = 0.03) and between the scores of neutral and joy (estimate = -2.13, *p* = 0.001), was greater in the children with ASD than in the TD controls. There were no significant differences between joy and the three other emotions (anger, fear, and sadness) for the ASD group and the control group. Finally, the discrepancy between the results for the unimodal visual stimuli and the bimodal stimuli was less important in the ASD group than in the control group (estimate = 0.61, *p* = 0.033), which suggests that adding audio for TD children was more useful than for children with ASD.

Eye-tracking data revealed that the ASD group made more saccades (estimate = 0.61, *p* = 0.002) and had shorter fixation durations (estimate = -0.3, *p* = 0.012) than the TD controls. However, the eye-tracking variables were not associated with the emotion recognition scores (for saccades: estimate = 0.0003, *p* = 0.60; for fixation duration: estimate = -0.37, *p* = 0.054).

### Multidimensional Analysis by Task

For the *unimodal visual task*, there was a significant correlation between the probability of success and the children’s developmental age; the older children had greater scores. However, we found that there was no effect of sex (estimate = 0.24, *p* = 0.50), and we did not find an association between the eye-tracking data (saccades and fixation durations) and the scores of the participants (estimate = -0.0005, *p* = 0.52, and estimate = -0.46, *p* = 0.12, respectively). The children with ASD tended to perform worse than the TD group on this task (estimate = -0.59, *p* = 0.06). Within the ASD group, joy was better identified than the other emotions, including sadness (estimate = -2.01, *p* = 0.0001), anger (estimate = -1.25, *p* = 0.02), fear (estimate = -1.46, *p* = 0.008), disgust (estimate = -1.85, *p* < 0.001) and neutral (estimate = -1.46, *p* = 0.007). Interestingly, in the ASD group, we found no correlation between the recognition scores and developmental age (estimate = 0.14, *p* = 0.18), the Vineland total score (estimate = 0.009, *p* = 0.52), or the presence of language or coordination disorders (all *p* > 0.07). For the TD group, joy was better identified than anger (estimate = -2.36, *p* = 0.002), fear (estimate = -2.66, *p* < 0.001), and sadness (estimate = -3.12, *p* < 0.001). In contrast, no significant differences were found between the recognition scores for joy and the scores for neutral (estimate = -0.73, *p* = 0.41) and disgust (estimate = -1.46, *p* = 0.07).

Regarding *auditory stimuli processing*, the ASD group did not perform significantly worse than the TD group (estimate = -0.08, *p* = 0.64). There was a significant effect of the children’s developmental age: the older children had better scores (estimate = 0.3, *p* < 0.001). For the ASD group, developmental age was strongly correlated with the probability of success in emotion recognition (estimate = 0.24, *p* < 0.001) and, to a lesser extent, the Vineland total score (estimate = 0.015, *p* = 0.02) and the presence of language impairment (estimate = -0.48, *p* = 0.05). With regard to specific emotions, the two groups obtained the lowest scores, with equivalent values, for anger.

Finally, during the *bimodal emotional task*, the ASD group did not perform significantly worse than the TD group (estimate = -0.62, *p* = 0.10). Joy was better recognized than anger (estimate = -1.62, *p* < 0.001) and neutral (estimate = -0.93, *p* = 0.03). These results were similar for the ASD group. The probability of success was strongly associated with the children’s developmental age: the older children had significantly higher scores (estimate = 0.6, *p* < 0.001). By contrast, we found that there was no significant association between the probability of success in the emotion recognition task and the Vineland total score or the presence of language or coordination disorders (all *p* > 0.51). Moreover, the probability of emotion recognition was not associated with the eye-tracking data.

## Discussion

Our study aimed to reveal the specific patterns of emotional processing in children with ASD (according to the PDD diagnostic category of the ICD-10), when compared to normally developing children, through a multimodal identification task and the assessment of developmental comorbidities. As a whole, the children with ASD performed well on the emotional multimodal tasks. Unlike Vannetzel et al.’s (2010) study, we did not find a significant difference between the scores that were obtained by the two groups. We also did not find a significant difference between the scores of the two groups on the auditory and bimodal tasks. The discrepancy between Vannetzel et al.’s (2010) results and our results may be explained by their sample, which included patients with PDD-NOS who also met the Multiple Complex Developmental Disorder criteria (i.e., patients also exhibited emotional/anxiety symptoms; Cohen et al., 1994; Buitelaar and Van der Gaag, 1998; Xavier et al., 2011). Similar to Jones et al. (2011), who tested adolescents with and without ASD by using facial and vocal emotional tasks, we found that the probability of correct emotion recognition was strongly correlated with the developmental age of the participants for each of the tasks. Interestingly, these results were confirmed in the ASD group, except for the visual task. In the ASD group, this may be explained by two factors: (1) for the unimodal visual modality, this group tends to perform worse than the TD group, but most of all, (2) the auditory modality seems to be discriminant in this association, with the strongest estimate between emotion recognition scores and developmental age being found for this task (estimate = 0.24, *p* < 0.001) and being higher than that of the bimodal task (estimate = 0.52, *p* < 0.001). However, Jones et al. (2011) described a similar emotion processing style in the ASD and non-ASD groups, except for a difficulty in recognizing surprise. On the contrary, we found, consistent with the literature, an important heterogeneity in our results, which could be considered to be a key issue.

For the entire multimodal task, we found a strong contrast between joy recognition (the emotion that was most easily identified) and the neutral emotion or anger (the most difficult to identify) for the ASD group. These results, which are congruent with the data of Vannetzel et al. (2010), are also found in the bimodal and auditory tasks’ secondary analyses. The results concerning the neutral emotion could be due to abnormalities in the recognition of specific emotions in ASD children, based on a greater discrepancy being found between neutral and joy recognition in the ASD group than the TD group. Conversely, for anger in the auditory task, both groups obtained the lowest scores, which had equivalent values.

The ASD group performed significantly worse than the TD group in the case of the visual and bimodal stimuli. The lowest scores were obtained by both of the groups in the auditory task; multisensory processing allowed children with ASD to partially compensate for the difficulties that were experienced in the visual modality, which confirms the results of Vannetzel et al. (2010). This result seems to be incongruent with the impairments in multimodal processing that are described in individuals with ASD (Happe and Frith, 2006; Collignon et al., 2013). However, according to Charbonneau et al. (2013), the discrepancy between the visual and bimodal tasks is significantly more important in the TD group than in the ASD group. These results do not necessarily contradict the “weak central coherence theory” in regard to people with ASD (Happe and Frith, 2006); this partial improvement in accuracy on the bimodal task could be better explained by the addition of redundant (visual + auditory) targets, rather than the multisensory integration of visual and auditory cues into a unified percept.

A few studies of young adults have demonstrated that congruent emotional information that is processed via multisensory channels optimizes behavioral responses, which results in enhanced accuracy and a faster response time (RT; see De Gelder and Vroomen, 2000; Kreifelts et al., 2007; Luherne-du Boullay et al., 2014). Multisensory enhancement is sometimes explored in behavioral studies that use RT by comparing the observed RT distribution to the distribution that is predicted by a ‘race model’ (e.g., Colonius and Diederich, 2006). Multisensory integration occurs when the reaction time for bimodal trials is faster than what is predicted by the race model (e.g., Charbonneau et al., 2013; Luherne-du Boullay et al., 2014). Testing this hypothesis by using RTs in our study was not possible because the RTs were not recorded due to the particular eye-tracking procedure (i.e., forced stimuli exploration over 3 s) and the studied population (i.e., TD children and children with ASD).

The eye-tracking data revealed interesting differences between the two groups (i.e., an important number of saccades and shorter fixation durations for the children with ASD than TD children). These results are consistent with studies that describe gaze abnormalities in ASD, including a shorter time being spent on emotional cues (for a review, see Guillon et al., 2014; Yi et al., 2014). However, these different gaze patterns were not associated with the scores of the participants and were not able to explain the discrepancy between the performances of the two groups. It is, therefore, difficult to conclude by stating that autistic children process faces in a holistic fashion. These abnormalities could be, in part, explained by our experimental design and the eye-tracking data analysis (see limitations).

Because children with DCD have poor cross-modal integration (see Wilson et al., 2013, for a meta-analysis) and frequent neuro-visual impairments, we expected that children with ASD and comorbid DCD would preferentially fail on the unimodal visual or bimodal visuo-auditory tasks. Our results were not consistent with this hypothesis; no association was found between the presence of a DCD and the scores on the visual or bimodal tasks. We assumed that children in the ASD group who had comorbid language disorders would be particularly impaired concerning unimodal auditory stimuli. Our hypothesis was partially correct: we found a statistical trend in this direction (estimate = -0.48, *p* = 0.05) regarding this modality. Furthermore, we did not find any association between the presence of this comorbidity and the scores for emotion recognition on the other tasks (visual and bimodal; all *p* > 0.78). Finally, a unique significant correlation between the scores on Vineland and the performances in emotion recognition was found for the auditory stimulus.

There are several limitations that warrant consideration. First, different metrics were used to match the ASD and TD groups based on age. Cognitive assessments were performed on the ASD group to assess developmental age but not on the TD group, for which chronological age was used instead. In addition, the lack of some significant effects may be due to the power of the study. Given the small sample size of our groups and considering the clinical heterogeneity of the ASD group, we were only able to detect major effect sizes and unable to detect possible subtle impacts of co-occurring factors (i.e., comorbidities) or potentially clinically meaningful effects (i.e., Vineland Adaptative Coordination Scale). Regarding the eye-tracking data analysis, we consider faces as a whole, rather than more precisely analyzing the gaze upon certain areas of interest (e.g., eyes, nose, and mouth). This choice is a limitation for the fine comparisons between children with ASD and TD children when exploring faces during an emotional task. However, our analysis was based on current, state-of-the-art machine learning methods to recognize facial emotion. Machine learning has shown that facial emotion classifiers use Action Units that are distributed all over the face to achieve the best performance (Sénéchal et al., 2012).

## Conclusion

Children with ASD demonstrate rather high performance on emotion recognition, particularly for multimodal stimuli. The difficulties that are experienced for visual stimuli are partially alleviated when using bimodal stimuli. Developmental age plays a major role for TD children, whereas its role is limited to the multimodal task for children with ASD. However, performances in emotion recognition in ASD are heterogeneous and do not simply correlate with comorbidities. The existence of a language disorder seems to have an impact on the performances of the ASD group in the auditory modality. Conversely, in the visual or bimodal (visuo-auditory) tasks, the impact of a DCD or gaze impairments has not been demonstrated. Future studies with larger samples will allow researchers to confirm and refine these data. Given the heterogeneity of the results that are found in the literature, we wonder whether impaired emotion processing may constitute a dimension that should be explored in ASD so that we can define subgroups in this condition that are more homogeneous and tailor interventions.

## Conflict of Interest Statement

The authors declare that the research was conducted in the absence of any commercial or financial relationships that could be construed as a potential conflict of interest.

## References

[B1] American Psychiatric Association (APA) (2000). *Diagnostic and Statistical Manual of Mental Disorders (DSM-IV-TR)*, 4th Edn Washington, DC: American Psychiatric Association.

[B2] Ashwin C. Chapman E. Colle L. Baron-Cohen S. (2006). Impaired recognition of negative basic emotions in autism: a test of the amygdala theory. *Soc. Neurosci.* 1 349–363. 10.1080/17470910601040772 18633799

[B3] Baker K. F. Montgomery A. Abramson R. (2010). Brief report: perception and lateralization of spoken emotion by youths with high-functioning forms of autism. *J. Autism Dev. Disord.* 40 123–129. 10.1007/s10803-009-0841-1 19653088

[B4] Bebko J. Weiss J. Demark J. Gomez P. (2006). Discrimination of temporal synchrony in intermodal events by children with autism and children with developmental disabilities without autism. *J. Child Psychol. Psychiatry* 47 88–98. 10.1111/j.1469-7610.2005.01443.x 16405645

[B5] Belin P. Fillion-Bilodeau S. Gosselin F. (2008). The montreal affective voices: a validated set of nonverbal affect bursts for research on auditory affective processing. *Behav. Res. Methods* 40 531–539. 10.3758/BRM.40.2.531 18522064

[B6] Braverman M. Fein D. Lucci D. Waterhouse L. (1989). Affect comprehension in children with pervasive developmental disorders. *J. Autism Dev. Disord.* 19 301–316. 10.1007/BF02211848 2745394

[B7] Buitelaar J. K. Van der Gaag R. J. (1998). Diagnostic rules for children with PDD-NO Sand multiple complex developmental disorder. *J. Child Psychol. Psychiatry* 39 911–919. 10.1017/S0021963098002820 9758199

[B8] Buitelaar J. K. Van der Wees M. Swaab-Barneveld H. Van der Gaag R. J. (1999). Theory of mind and emotion-recognition functioning in autistic spectrum disorders and in psychiatric control and normal children. *Dev. Psychopathol.* 11 39–58. 10.1017/S0954579499001947 10208355

[B9] Castelli F. (2005). Understanding emotions from standardized facial expressions in autism and normal development. *Autism* 9 428–449. 10.1177/1362361305056082 16155058

[B10] Chaby L. Chetouani M. Plaza M. Cohen D. (2012). “Exploring multimodal social-emotional behaviors in autism spectrum disorders: an interface between social signal processing and psychopathology,” in *Proceedings of the International Conference on Privacy, Security, Risk and Trust and 2012 International Conference on Social Computing*, Washington, DC: IEEE Computer Society, 950–954. 10.1109/SocialCom-PASSAT.2012.111

[B11] Charbonneau G. Bertone A. Lepore F. Nassim M. Lassonde M. Mottron L. (2013). Multilevel alterations in the processing of audio-visual emotion expressions in autism spectrum disorders. *Neuropsychologia* 51 1002–1010. 10.1016/j.neuropsychologia.2013.02.009 23462241

[B12] Cohen D. J. Towbin K. E. Mayes L. Volkmar F. (1994). “Developmental psychopathology of multiplex developmental disorder,” in *Developmental Follow-up: Concepts, Genres, Domains, and Methods*, eds Friedman S. L. Haywood H. C. (New York, NY: Academic Press Inc), 155–179.

[B13] Cohen J. (1988). *Statistical Power Analysis for the Behavioral Sciences.* Hillsdale, NJ: Lawrence Erlbaum Associates.

[B14] Collignon O. Charbonneau G. Peters F. Nassim M. Lassonde M. Lepore F. (2013). Reduced multisensory facilitation in persons with autism. *Cortex* 49 1704–1710. 10.1016/j.cortex.2012.06.001 22818902

[B15] Colonius H. Diederich A. (2006). The race model inequality: interpreting a geometric measure of the amount of violation. *Psychol. Rev.* 113 148–154. 10.1037/0033-295X.113.1.148 16478305

[B16] Corden B. Chilvers R. Skuse D. (2008). Avoidance of emotionally arousing stimuli predicts social-perceptual impairment in Asperger’s syndrome. *Neuropsychologia* 46 137–147. 10.1016/j.neuropsychologia.2007.08.005 17920642

[B17] De Gelder B. Vroomen J. (2000). The perception of emotions by ear and by eye. *Cogn. Emot.* 14 289–311. 10.1080/026999300378824

[B18] de Wit T. C. Falck-Ytter T. von Hofsten C. (2008). Young children with autism spectrum disorder look differently at positive versus negative emotional faces. *Res. Autism Spectr. Disord.* 2 651–659. 10.1016/j.rasd.2008.01.004

[B19] Downs A. Smith T. (2004). Emotional understanding, cooperation, and social behavior in high-functioning children with autism. *J. Autism Dev. Disord.* 34 625–635. 10.1007/s10803-004-5284-0 15679183

[B20] Ebner N. Riediger M. Lindenberger U. (2010). Faces - a database of facial expressions in young, middle-aged, and older women and men: development and validation. *Behav. Res. Methods* 42 351–362. 10.3758/BRM.42.1.351 20160315

[B21] Grossman J. B. Klin A. Carter A. S. Volkmar F. (2000). Verbal bias in recognition of facial emotions in children with Asperger syndrome. *J. Child Psychol. Psychiatry* 41 369–379. 10.1111/1469-7610.00621 10784084

[B22] Guillon Q. Hadjikhani N. Baduel S. Rogé B. (2014). Visual social attention in autism spectrum disorder: insights from eye tracking studies. *Neurosci. Biobehav. Rev.* 42 279–297. 10.1016/j.neubiorev.2014.03.013 24694721

[B23] Happe F. Frith U. (2006). The weak coherence account: detail-focused cognitive style in autism spectrum disorders. *J. Autism Dev. Disord.* 36 5–25. 10.1007/s10803-005-0039-0 16450045

[B24] Harms M. Martin A. Wallace G. (2010). Facial emotion recognition in autism spectrum disorders: a review of behavioral and neuroimaging studies. *Neuropsychol. Rev.* 20 290–322. 10.1007/s11065-010-9138-6 20809200

[B25] Henderson S. E. Sugden D. A. (1992). *Movement Assessment Battery for Children.* London: Psychological Corp.

[B26] Humphreys K. Minshew N. Leonard G. L. Behrmann M. (2007). A fine-grained analysis of facial expression processing in high-functioning adults with autism. *Neuropsychologia* 45 685–695. 17010395 10.1016/j.neuropsychologia.2006.08.003

[B27] Jones C. R. G. Pickles A. Falcaro M. Marsden A. J. S. Happé F. Scott S. K. (2011). A multimodal approach to emotion recognition ability in autism spectrum disorders. *J. Child Psychol. Psychiatry* 52 275–285. 10.1111/j.1469-7610.2010.02328.x 20955187

[B28] Kanner L. (1943). Autistic disturbances of affective contact. *Nerv. Child* 2 217–250. 4880460

[B29] Khomsi A. (2001). *Evaluation du Langage Oral.* Paris: ECPA.

[B30] Kreifelts B. Ethofer T. Grodd W. Erb M. Wildgruber D. (2007). Audiovisual integration of emotional signals in voice and face: an event-related fMRI study. *Neuroimage* 37 1445–1456. 10.1016/j.neuroimage.2007.06.020 17659885

[B31] Lacroix A. Guidetti M. Rogé B. Reilly J. (2009). Recognition of emotional and nonemotional facial expressions: a comparison between Williams syndrome and autism. *Res. Dev. Disabil.* 30 976–985. 10.1016/j.ridd.2009.02.002 19286347

[B32] Lord C. Rutter M. Le Couteur A. (1994). Autism diagnostic interview revised: a revised version of a diagnostic interview for caregivers of individuals with possible pervasive developmental disorders. *J. Autism Dev. Disord.* 24 659–685. 10.1007/BF02172145 7814313

[B33] Luherne-du Boullay V. Plaza M. Perrault A. Capelle L. Chaby L. (2014). Atypical crossmodal emotional integration in patients with gliomas. *Brain Cogn.* 92 1–16. 10.1016/j.bandc.2014.10.003 25463143

[B34] Magnée M. de Gelder B. van England H. Kemner C. (2008). Audiovisual speech integration in pervasive developmental disorder: evidence from event-related potentials. *J. Child Psychol. Psychiatry* 49 995–1000. 10.1111/j.1469-7610.2008.01902.x 18492039

[B35] Megnin O. Flitton A. Jones C. R. G. de Haan M. Baldeweg T. Charman T. (2012). Audiovisual speech integration in autism spectrum disorders: ERP evidence for atypicalities in lexical-semantic processing. *Autism Res.* 5 39–48. 10.1002/aur.231 22162387 PMC3586407

[B36] Mongillo E. Irwin J. Whalen D. Klaiman C. Carter A. Schultz R. (2008). Audiovisual processing in children with and without autism spectrum disorders. *J. Autism Dev. Disord.* 38 1349–1358. 10.1007/s10803-007-0521-y 18307027

[B37] Nuske H. J. Vivanti G. Dissanayake C. (2013). Are emotion impairments unique to, universal, or specific in autism spectrum disorder? A comprehensive review. *Cogn. Emot.* 27 1042–1061. 10.1080/02699931.2012.762900 23387530

[B38] Nuske H. J. Vivanti G. Dissanayake C. (2014). Reactivity to fearful expressions of familiar and unfamiliar people in children with autism: an eye-tracking pupillometry study. *J. Neurodev. Disord.* 6:14 10.1186/1866-1955-6-14 PMC4064262 24982695

[B39] Ozonoff S. Pennington B. Rogers S. (1990). Are there emotion perception deficits in young autistic children? *J. Child Psychol. Psychiatry* 31 343–361. 10.1111/j.1469-7610.1990.tb01574.x 2318918

[B40] Pelphrey K. A. Sasson N. J. Reznick J. S. Paul G. Goldman B. D. Piven J. (2002). Visual scanning of faces in autism. *J. Autism Dev. Disord.* 32 249–261. 10.1023/A:1016374617369 12199131

[B41] Philip R. C. M. Whalley H. C. Stanfield A. C. Sprengelmeyer R. Santos I. Young A. W. (2010). Deficits in facial, body movement and vocal emotional processing in autism spectrum disorders. *Psychol. Med. J. Res. Psychiatry Allied Sci.* 40 1919–1929. 10.1017/S0033291709992364 20102666

[B42] Prior M. R. Dahlstrom B. Squires T. L. (1990). Autistic children’s knowledge of thinking and feeling states in other people. *J. Child Psychol. Psychiatry* 31 587–601. 10.1111/j.1469-7610.1990.tb00799.x 2365761

[B43] Russo N. Mottron L. Burack J. A. Jemel B. (2012). Parameters of semantic multisensory integration depend on timing and modality order among people on the autism spectrum: evidence from event-related potentials. *Neuropsychologia* 50 2131–2141. 10.1016/j.neuropsychologia.2012.05.003 22613013

[B44] Sawyer A. C. P. Williamson P. Young R. L. (2012). Can gaze avoidance explain why individuals with Asperger’s syndrome can’t recognize emotions from facial expressions? *J. Autism Dev. Disord.* 42 606–618. 10.1007/s10803-011-1283-0 21590432

[B45] Sénéchal T. Rapp V. Salam H. Seguier R. Bailly K. Prevost L. (2012). Facial action recognition combining heterogeneous features via multi-kernel learning. *IEEE Trans. Syst. Man Cybern. Part B* 42 993–1005. 10.1109/TSMCB.2012.2193567 22623430

[B46] Sinzig J. Morsch D. Lehmkuhl G. (2008). Do hyperactivity, impulsivity and inattention have an impact on the ability of facial affect recognition in children with autism and ADHD? *Eur. Child Adolesc. Psychiatry* 17 63–72. 10.1007/s00787-007-06379 17896119

[B47] Sparrow S. Balla D. Achetti D. (1984). *Vineland Adaptative Behaviour Scales, Articles Rines MN.* Englewood, CO: American Guidance Service.

[B48] Uljarevic M. Hamilton A. (2013). Recognition of emotions in autism: a formal meta-analysis. *J. Autism Dev. Disord.* 43 1517–1526. 10.1007/s10803-012-1695-5 23114566

[B49] Vannetzel L. Chaby L. Cautru F. Cohen D. Plaza M. (2010). Neutral versus emotional human stimuli processing in children with pervasive developmental disorders not otherwise specified. *Res. Autism Spectr. Disord.* 5 775–783. 10.1016/j.rasd.2010.09.005

[B50] Wallace S. Coleman M. Bailey A. (2008). An investigation of basic facial expression recognition in autism spectrum disorders. *Cogn. Emot.* 22 1353–1380. 10.1080/02699930701782153

[B51] Wilson P.-H. Ruddock S. Smits-Engelsman B. Polatajko H. Blank R. (2013). Understanding performance deficits in developmental coordination disorder: a meta-analysis of recent research. *Dev. Med. Child Neurol.* 55 217–228. 10.1111/j.1469-8749.2012.04436.x 23106668

[B52] World Health Organization (1993). *The ICD-10 Classification of Mental and Behavioural Disorders: Diagnostic Criteria for Research.* Geneva: World Health Organization.

[B53] Xavier J. Bursztejn C. Stiskin R. Canitano R. Cohen D. (2015). Autism spectrum disosrders: a developmental and dimensional approach toward a tailored therapeutic program. *Res. Autism Spectr. Disord.* 18 21–33. 10.1016/j.rasd.2015.06.011

[B54] Xavier J. Vannetzel L. Viaux S. Leroy A. Plaza M. Tordjman S. (2011). Reliability and diagnostic efficiency of the diagnostic inventory for disharmony (DID) in youths with pervasive developmental disorder and multiple complex developmental disorder. *Res. Autism Spectr. Disord.* 5 1493–1499. 10.1016/j.rasd.2011.02.010

[B55] Yi L. Feng C. Quinn P. C. Ding H. Li J. Liu Y. (2014). Do individuals with and without autism spectrum disorder scan faces differently? A new multi-method look at an existing controversy. *Official J. Int. Soc. Autism Res.* 7 72–83. 10.1002/aur.1340 24124133

